# Exploiting implicit social relationships via dimension reduction to improve recommendation system performance

**DOI:** 10.1371/journal.pone.0231457

**Published:** 2020-04-22

**Authors:** Ali M. Ahmed Al-Sabaawi, Hacer Karacan, Yusuf Erkan Yenice

**Affiliations:** 1 Department of Electrical Electronic and Computer Engineering, Faculty of Engineering, Aksaray University, Aksaray, Turkey; 2 Department of Computer Science, College of Computer Sciences and Mathematics, Mosul University, Mosul, Iraq; 3 Department of Computer Engineering, Faculty of Engineering, Gazi University, Ankara, Turkey; University of Konya Technical, TURKEY

## Abstract

The development of Web 2.0 and the rapid growth of available data have led to the development of systems, such as recommendation systems (RSs), that can handle the information overload. However, RS performance is severely limited by sparsity and cold-start problems. Thus, this paper aims to alleviate these problems. To realize this objective, a new model is proposed by integrating three sources of information: a user-item matrix, explicit and implicit relationships. The core strategy of this study is to use the multi-step resource allocation (MSRA) method to identify hidden relations in social information. First, explicit social information is used to compute the similarity between each pair of users. Second, for each non-friend pair of users, the MSRA method is applied to determine the probability of their relation. If the probability exceeds a threshold, a new relationship will be established. Then, all sources are incorporated into the Singular Value Decomposition (SVD) method to compute the missing prediction values. Furthermore, the stochastic gradient descent technique is applied to optimize the training process. Additionally, two real datasets, namely, Last.Fm and Ciao, are utilized to evaluate the proposed method. In terms of accuracy, the experiment results demonstrate that the proposed method outperforms eight state-of-the-art approaches: Heats, PMF, SVD, SR, EISR-JC, EISR-CN, EISR-PA and EISR-RAI.

## Introduction

The amount of available data is growing rapidly and it is extremely complicated for users to find their preferences in this huge amount of data. In addition, the increased use of technology in our lives has boosted peoples’ demand for intelligent systems that execute this task, which are called recommendation systems (RSs). The main objective of a RS is to predict items that might be of interest (e.g., movies, music, books, and newspapers) to users based on enormous amounts of data [1, 2]. A basic RS relies on several technologies, such as information filtering, user modelling, and classification learning [3]. Typical recommendation systems include collaborative filtering systems (CFs), content-based systems (CBs), and hybrid systems [4]. A CF learns a user-item matrix for recommending items. A CB utilizes users’ properties to identify new items [5]. A hybrid system integrates CF and CB to recommend items. CFs are the most prevalent type of RSs and can be further subdivided into memory-based and model-based systems. A memory-based system computes the similarity of users/items to find the nearest *n* users/items for recommendation. The neighbour-model CF is a highly diffuse method of this type that depends on human ratings as the main feedback; the recommended items are identified by computing the similarities between users. By contrast, a model-based system learns to recommend items to users by dividing the dataset into two parts (training and testing) and applying machine learning techniques.

Many methods of this type have been proposed, such as clustering CF and matrix factorization (MF) [6, 7]. MF has become a very prevalent method in RSs since it produces highly accurate results and can reduce the user-item matrix to a small number of latent factors, from which the attitude of the user in the system can be extracted. Many approaches use CFs to predict items for users. The most prevalent methods are dimension reduction methods, such as SVD, Probabilistic Matrix Factorization (PMF), and non-negative matrix factorization (NMF). In this study, SVD is applied to predict items, which identifies the hidden information in the data source. However, RSs face challenges that have negative effects on the accuracy. Moreover, the huge size of the data, which reaches millions of items in websites such as Netflix, increases the sparsity inside the dataset. Therefore, the accuracy of the recommendation effected passively. This problem called sparsity [8]. Another issue is the cold-start problem, which refers to the problem of making predictions for a new customer or item: The RS cannot recommend items to a new user because there is no history regarding his/her tastes. Likewise, the RS lacks information regarding the rate or score of a cold-start item [9]. Cold-start is divided into two types: incomplete cold start (ICS) and complete cold start (CCS). ICS refers to the scenario in which the cold-start user/item has poor feedback (3 ratings or less), whereas CCS refers to the scenario in which the user/item does not have any score and, hence, the RS lacks relevant items/users [10].

The main elements in an RS are users, items and rating values; these elements constitute a user-item matrix. The traditional methods utilize these elements as basic knowledge to recommend preference items to users [11, 12]. However, the results are inaccurate since the growth in the amount of data worsens the problems that are discussed above. In response, researchers tend to use social information in RSs since the social information provides an additional source of data [3, 13–15].

Recently, the use of social networks to enhance recommendation accuracy has attracted many researchers in recommendation systems. They applied explicit relations directly [3, 6, 16–18]. The main factor of the social network information is the explicit friends: the recommendation system harnesses the preferences of the user’s friends to explore new items. However, the sparsity and accuracy problems are still encountered. Previous studies assumed that connected users have almost the same preferences, whereas unconnected users have different tastes. In practice, users have similar preferences, even users who are far away in the network. This type of relation is called an implicit relation [18–21]. Implicit relations increase the prediction accuracy by alleviating the sparsity problem. Many methods are applied to infer new relationships in a social dataset. These methods realize the objective of predicting new relationships among explicit relations and they enhance the prediction accuracy. However, these methods depend only on the direct node neighbours and ignore the undirected nodes, which may lead to the failure to consider vital information.

In this paper, a new method is proposed for predicting implicit relationships, namely, multi-step resource allocation (MSRA), which can overcome the limitations of the previous methods. MSRA is integrated with the other two sources (the explicit relationships and the user-item matrix) into the dimension reduction method to produce a new model. The remainder of this paper is organized as follows: Section 2 discusses related work. Section 3 describes the methods that are utilized in the proposed method. In Section 4, the proposed method is presented. The experimental results are explained in Section 5. Finally, Section 6 presents the conclusions of this work.

## Related work

Recommendation systems (RSs) aim at providing items of interest to users. Many studies have been conducted on realizing this objective. However, one of the most widespread challenges is accuracy. The main causes of low accuracy are sparsity and cold-start. As the data size grows rapidly, users cannot rate most of the items, which results in data sparsity. Cold-start is an additional recommendation system problem, in which the user does not rate items and, hence, the system cannot distinguish the user’s preferences. Both negatively affect the performance of the recommendation system. To mitigate these problems, many studies have been conducted on all RS types.

A traditional recommendation system involves users, items, and transactions between users and items. A user-item rating matrix is used as a knowledge source of the RS [9]. Some RS methods employ users’ ratings as the only source of information and they do not consider any additional information. Most of these methods use expert ratings, user-generated feedback, and crowdsourcing (popularity) to produce a list of recommended items for each user [22–26]. Recently, in addition to rating information, modern RSs have used many information sources such as social information, forums, and social bookmarking [13]. The modern Web 2.0 provides the opportunity for users to establish their own relationships to create social networks. Thus, RSs can utilize these relationships as an extra information source to predict items [27]. In practice, people are influenced the tastes of their friends. For example, when someone is deciding whether buy, watch or listen to an item, he/she will check which items have been chosen by his/her friends. Therefore, social network information is useful in RSs. Many studies have used social network information as a supporting factor to alleviate the problems of RSs and to boost the recommendation performance [15, 28]. Social networks are satisfactory information sources for tackling RS problems; hence, many researchers have utilized social network information to enhance the performances of RSs [3, 15, 28]. In a social network, the relationships can be classified into two types: friend relationships and trust relationships.

Friend relationships, which are represented as an undirected graph, are mutual interactions between users in a social network where the RSs permit to the customers to establish friend relationships. By contrast, trust relationships (which are represented as a directed graph), are one-side relations; for example, user 1 trusts user 2 but user 2 does not necessarily trust user 1. In a trust relationship, some RSs allow users to specify trust values for other users. Various related works incorporate social networks into matrix factorization to improve the RS performance. Zheng et al. [15] proposed a regularization-based method that utilizes matrix factorization with social information as a factor. They incorporated two social regularizations: friendship relations and the correlations between users and items. Guo et al. [29] proposed a method that used transitions in trust relationships to identify new friends, which can improve the performance of the RS. Other researchers, such as Feng et al. [30], used social network information in the typically way, but they enhanced the prediction accuracy by increasing the number of learning loops.

Two social recommendation methods are proposed by Ma et al. [31]: average-based regularization and individual-based regularization (SR1 and SR2). In this study, both types of regularization are incorporated into the matrix factorization to compute the prediction of a missing rating. In the two methods, the authors used an explicit relation and they integrated it into the matrix factorization method. The results demonstrated that the SR model is more accurate than the state-of-the-art models, such as user-mean, item-mean, and NMF. Reafee et al. [32] proposed a new model (EISR) that exploited both the implicit social relationships and the explicit relationships to enhance the recommendation performance. The authors used link prediction to discover implicit relations, computed the similarity between the user and each new link, and integrated both explicit and implicit relations into PMF. However, the computing of the implicit relations is the main limitation of this method, for which they used the resource allocation index (RAI) to find implicit relations and to maintain reasonable accuracy comparing with many other similarity methods [21].

Link prediction (LP) techniques are used to discover new relationships from the explicit relations. LP refers to the prediction of new edges in the case of missing edges, for which many techniques are used. LP is utilized in many social network datasets, such as scientific co-authorship networks [18], biological interaction networks [33, 34], and e-commerce RSs [35, 36]. Reafee et al. [32] used the RAI as a similarity technique of LP to find new destination resources. However, it ignores multi-step neighbours in the search for other resources that may contain substantial information. Wu and Li [37] proposed MSRA, which can fuse the information of multi-step neighbours to convey a resource from the first neighbour level to another. In this study, LP is employed using the MSRA similarity technique. The primary reasons for utilizing LP are its ability to find the hidden relations of the network structure and that it does not require any additional information.

After reviewing the research that has been conducted in previous studies, we conclude that the traditional methods still lack sufficient prediction accuracy. This paper aims to increase the accuracy. Therefore, the proposed model addresses the following research questions (RQs):

RQ1: Can multi-step resource allocation be used instead of the resource allocation index to predict more implicit relationships with satisfactory accuracy?
RQ2: Does the incorporation of social information (explicit and implicit relations) with rating feedback information into the SVD method improve the performance of the RS?

## Materials and methods

A social network and dimension reduction are used to enhance the RS performance. To extract hidden information from the social network, a link prediction technique and a dimension reduction technique are used to explore the meaningful information from the user-item matrix via SVD. The following subsections include reviews of the link prediction and social network information.

### Link prediction

Link prediction is the task of predicting information that is missing from a social network. Three types of methods are utilized: link prediction based on features, link prediction based on the structure and a hybrid of the first two types. Many studies utilize methods of these three types [38, 19]. All these studies have attempted to improve the link prediction performance; however, the node characteristics are not always accessible. In this study, link prediction based on the structure is utilized to extract implicit friendships in a social network.

In the social network structure, a graph is used in which the nodes and edges correspond to the users and the relations, respectively. The social network is the main aspect in determining implicit friends. Therefore, many methods are used to predict new links, such as the Jaccard coefficient [17], common neighbours [18], preferential attachment [20] and the resource allocation index [21]. All these algorithms are implemented by finding the direct common friends of x and y, where x and y are non-friends (not connected in the social graph) and computing the similarity between x and y via the following formulas:

JaccardCoefficientsim(x,y)=(|ᴦ(x)⋂ᴦ(y)|)/(|ᴦ(x)∪ᴦ(y)|) (1)

$$Common\;Neighbors\;sim(x,y)=\frac{1}{k}(\left|ᴦ(x)⋂ᴦ(y)\right|)$$ (2)

$$Preferential\;Attachment\;sim(x,y)=\frac{1}{k}(\left|ᴦ(x)•ᴦ(y)\right|)$$ (3)

$$Resource\;Allocation\;Index\;sim(x,y)=\sum_{z\in \left|ᴦ(x)⋂ᴦ(y)\right|}\frac{1}{|ᴦ\left(z\right)|}$$ (4)

where *x* and *y* are the two users; ᴦ(*x*) and ᴦ(*y*) represent the sets of the friends of *x* and *y*, respectively; and *K* is the normalization factor.

As discussed in previous sections, RAI uses the first level of the common neighbours and ignores the other neighbours, which may lead to the failure to consider vital information. Wu and Li [37] proposed MSRA which can facilitate the process of identifying the common neighbours by finding undirected nodes between the targeted nodes. The authors used two-step resource transmission as MSRA_2_, as expressed in Eq (5), and three-step transmit resource transmission as MSRA_3_. In this work, MSRA_2_ is used to transmit resources and to predict new relationships.

$$s_{xy}^{MSRA2}=s_{xy}^{MSRA1}+\sum_{z1\in Z1}\sum_{z2\in Z2}\frac{1}{k_{z1}}\frac{1}{k_{z2}}$$ (5)

Where Z1 refers to ᴦ(*x*)∩ ᴦ(*y*) and Z2 to ᴦ(*z*1)∩ ᴦ(*y*)/*Z*1.

### Singular Value Decomposition (SVD)

SVD is a method of matrix factorization. In an RS, the user-item matrix can be decomposed into a low-dimensional matrix via SVD. The decomposition process extracts the patterns of various factors from the original user-item matrix. SVD can alleviate the sparsity problem by constructing a low-dimensional matrix. SVD was used for collaborative filtering in the Netflix competition to realize the objectives of recommendation algorithms [9]. Sarwar et al. [39] proposed a method in which the score of the prediction is computed after the dimension of movie data has been decreased via SVD. SVD reduces the rating scores of users and items to a specified dimension by extracting the latent factors of both the users and the items [40]. For example, consider a 2-D user-item matrix (M), of which the rows correspond to the users (u) and the columns correspond to the items (i). This matrix can be decomposed into three matrices as M = U_u*k_ • S_k*k_ • V_i*k_, where *u* is the number of users, *i* the number of items and *k* is the dimension. U and V are orthogonal matrices; hence, the eigenvectors are associated with *k* nonzero eigenvalues. S is a diagonal matrix that has *k* nonzero values. U is the eigenvector matrix that is identical to the rows of the original matrix. V is an eigenvector matrix that corresponds to the columns of the original matrix. S is a diagonal matrix in which the eigenvalues are in descending order from the most important information to the least important. As the dimension of S decreases, the sizes of U and V also decrease. In addition, the original matrix contains too much information and a portion of this information is noise. Thus, by reducing the dimension to a specified value, the noise will be removed as well. SVD can find the best low-rank linear representation of the user-item matrix and can remove the noise from the data. One type of noise is due to users rating the items randomly; ratings of this type are not useful for determining whether an item is of interest. Finally, the prediction is conducted by multiplying the user vector by the item vector and summing them with the baseline.

$$SVD(A)=U*S*V^{T}$$ (6)

$$\bar{r_{ui}}=b_{ui}+V_{i}^{T}U_{u}$$ (7)

where *b*_*ui*_ represents the baseline predictor for the unknown items, which can be computed by:

$$b_{ui}=\mu +b_{u}+b_{i}$$ (8)

in which *μ* denotes the mean of all the data and *b*_*u*_ and *b*_*i*_ refer to the observed deviations of user *u* and item *i*, respectively, which are computed from the mean value. The stochastic gradient descent optimization algorithm can be used to update the values of the factors (U, V, b_u_, and b_i_) by reducing the regularization square error as expressed in the following objective formula:

$$E=\sum_{(u,i)\in m}{\left(r_{ui}-\mu -b_{u}-b_{i}-V_{i}^{T}U_{u}\right)}^{2}+\lambda ({b_{i}}^{2}+{b_{u}}^{2}+{\left\|V\right\|}^{2}+{\left\|U\right\|}^{2})$$ (9)

Then, new values are recovered for all updated factors. The learning rules are expressed in the following formulas:

$$b_{u}=b_{u}+\alpha \;(E-\lambda b_{u})$$ (10)

$$b_{i}=b_{i}+\alpha \;(E-\lambda b_{i})$$ (11)

$$U_{u}=U_{u}+\alpha \;(E\;V_{i}-\lambda U_{u})$$ (12)

$$V_{i}=V_{i}+\alpha \;(E\;U_{u}-\lambda V_{i})$$ (13)

in which *λ* is a constant value that is used to regularize the factors and to avoid over-fitting, ‖*x*‖ denotes the Frobenius norm, and *α* is the learning rate.

### Social regularization

With the development of Web 2.0 and the construction of relationships in many applications, social networks have become important resources for supporting RSs in recommending new items for users. Friend relationships play a major role in RSs, as users are influenced by their friends regarding everything they want to buy. Ma et al. [31] proposed two types of social regularization (SR): average-based regularization and individual-based regularization. In this study, both types of regularization combine social information with matrix factorization to predict missing ratings. According to their results, the SR model is more accurate than state-of-the-art models such as user-mean, item-mean, NFM, and PMF. The objective function of the individual model defined as

$$E=\frac{1}{2}\sum_{i=1}^{m}\sum_{j=1}^{n}I_{ij}{\left(R_{ij}-U^{T}V\right)}^{2}+\frac{\lambda_{u}}{2}{\left\|U\right\|}_{Fro}^{2}+\frac{\lambda_{v}}{2}{\left\|V\right\|}_{Fro}^{2}+\frac{\beta_{e}}{2}\sum_{i=1}^{m}\sum_{f\epsilon F(i)}sim(i,f){\left\|U_{i}-U_{f}\right\|}_{fro}^{2}$$ (14)

where *β*_*e*_ > 0 is the factor that controls the degree of similarity of the explicit relationships for social regularization, *sim*(*i*,*f*) computes the similarity between user *i* and his/her friend *f*, and *F(i)* denotes the set of the explicit friends of user *i*. This model assumes that the user is influenced by the tastes of his/her explicit friends; therefore, the degrees of similarity between the user and his/her friends are used to increase the accuracy of the recommendation. However, using explicit relationships is not sufficient since the social network grows rapidly and the number of users increases simultaneously; therefore, the system requires more factors for increasing the accuracy of the recommendation. Reafee et al. [32] proposed a new model (EISR) that exploited both implicit social relationships and explicit relationships to enhance the recommendation performance. The authors used link prediction to discover an implicit relation and computed the similarity between the user and the new link, which is added as a new factor into the objective function of Eq (14), which must be updated by integrating both the explicit and implicit relations as follows:

$$E=\frac{1}{2}\sum_{i=1}^{m}\sum_{j=1}^{n}I_{ij}{\left(R_{ij}-g(U^{T}V)\right)}^{2}+\frac{\lambda_{u}}{2}{\left\|U\right\|}_{Fro}^{2}+\frac{\lambda_{v}}{2}{\left\|V\right\|}_{Fro}^{2}+\frac{\beta_{e}}{2}\sum_{i=1}^{m}\sum_{f\epsilon F(i)}sim(i,f){\left\|U_{i}-U_{f}\right\|}_{fro}^{2}+\frac{\beta_{i}}{2}\sum_{i=1}^{m}\sum_{f^{*}\epsilon F^{*}(i)}sim(i,f^{*}){\left\|U_{i}-U_{f^{*}}\right\|}_{fro}^{2}$$ (15)

where *g(x)* is the logistic function *g(x) = 1 / (1+ exp (-x))*, f* represents the implicit relation, F*(*i*) denotes the set of the implicit friends of user *i*, *β*_*i*_ > 0 is the parameter for restricting the adversity of the explicit and implicit relations, and *sim*(*i*,f*) is the similarity between user *i* and his/her implicit friends.

Both SR and EISR succeeded in enhancing the accuracy of the prediction. However, in EISR, four approaches were used to identify the implicit relations and all these methods considered the similarity with the direct common neighbours in each non-friend pair, whereas the undirected neighbours, which may contain rich information, were eliminated. In this study, MSRA is applied as a link prediction technique to extract the implicit relationships and explicit relationships are incorporated via the SVD method to alleviate the sparsity and incomplete cold-start problems.

## Proposed method

**Fig 1:**
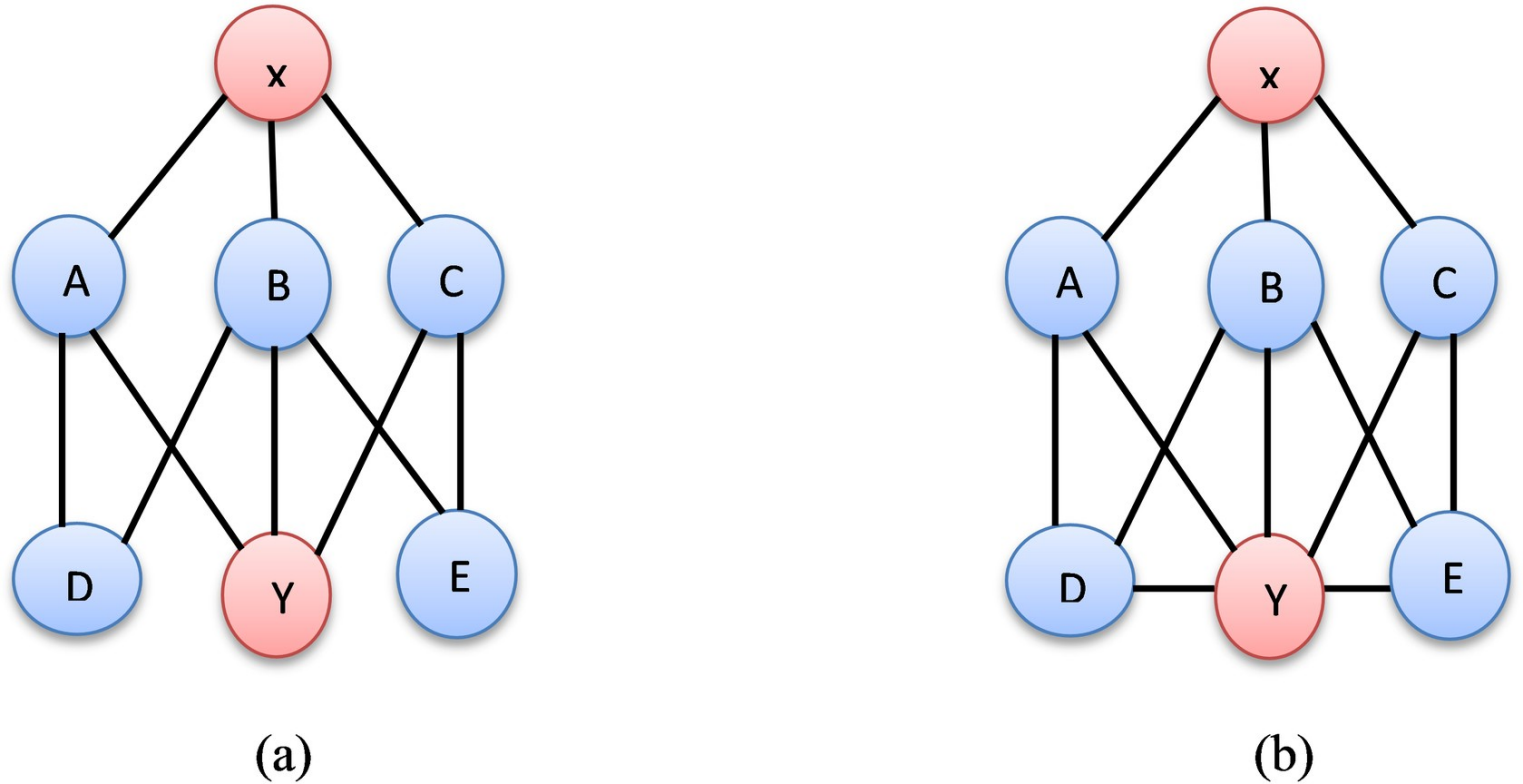
Social network nodes.

The proposed method includes three information resources, namely, a user-item matrix, an explicit relation, and an implicit relation, which are obtained using the MSRA method that was proposed by Wu and Li [37]. The first two resources are available directly from the dataset, whereas the MSRA is computed from explicit relationships. For instance, consider two unconnected nodes, namely, *x* and *y*. The lists of friends for both nodes should be determined. The similarity of the common neighbours for both nodes is computed via inverse summation. If the value exceeds a threshold, a new relationship will be created. (Fig 1) presents an example of computing MSRA in social network nodes. In this figure, *x* and *y* are unconnected nodes and the common neighbours between them are A, B, and C. By using RAI, the similarity is computed by calculating 1/3 + 1/4 + 1/3 = 0.92 since nodes A and C have 3 relationships each and B has 4 relationships. In Fig 1A and 1B, we obtain the same result using RAI because it depends only on the directed connections between the targeted nodes. However, in Fig 1B, there is more than one path from node *x* to node *y* through nodes A, B, and C; thus, the score of Fig 1B, should be higher than the score of Fig 1A. MSRA with two steps computes the score of Fig 1B as follows: first, it computes the score for the direct neighbours of *x* by applying the same approach as in RAI, multiplying the number of neighbours of node A with the number of neighbours of node D (since node D is a second-step neighbour of node x), namely, 1/3 *1/3, and multiplying the remaining nodes in the same way; the full computation is 1/3 * 1/3 + 1/4 * 1/3 +1/4 * 1/3 + 1/3 * 1/3 = 0.39. Then, the result is summed with the value that was computed previously (0.92). This method increases the scores between unconnected nodes. Subsequently, it increases the number of relationships, as demonstrated in the previous example. Now, the implicit relations of nodes *x* and *y* are A, B, C, D and F rather than A, B and C, as computed using RAI. The MSRA algorithm is presented as Algorithm 1.

Algorithm1: computing MSRA

Input: social array, threshold

Output: MSRA array

Index = 1

Foreach user i in social array

    Foreach user j in social array

        If i non-friend with j

            Foreach user k in social array

                If i friend k and j friend k

      Foreach user t in social array

        If j friend t and k friend t

            s1 = no. of friends of each k

            s2 = no. of friends of each t

            m = (1/s1) * (1/s2)

        endif

      endfor

      s = s + m

      endif

     endfor

        temp array(index) = s

     index = index + 1

    endif

   endfor

endfor

index = 1

foreach user i in social array

    for each user j in social array

        if i non-friend j

            foreach user k in social array

            If i friend k and j friend k

                z = no. of friends for each k

                s = s + (1/each z)

            endif

        endfor

        temp array(index) = temp array(index) + s

                if temp array(index) > threshold

            msra(n) = temp array(index)

            n = n + 1

        endif

        index = index + 1

    endif

endfor

endfor

After acquiring implicit relationships, each user has two groups of friends: explicit friends that are available directly in the social information data and the implicit friends that are extracted using MSRA method. subsequently, Pearson Correlation Coefficient method is exploited to compute the similarity between explicit friends and implicit friends by measuring the common rated items for both users as follow.

$$sim(i,f)=\frac{\sum_{h=1}^{n}\left(r_{i,jh}-\bar{r_{i}})(r_{f,jh}-\bar{r_{f}}\right)}{\sqrt{\sum_{h=1}^{n}{\left(r_{i,jh}-\bar{r_{i}}\right)}^{2}}\sqrt{\sum_{h=1}^{n}{\left(r_{f,jh}-\bar{r_{f}}\right)}^{2}}}$$ (16)

Where, *r*_*i*,*j*_ is the rating of item *j* by user *i*, *r*_*f*,*j*_ represents the rating of item *j* by user *f* and *f* indicates the friends of *i*. $\bar{r_{x}}$ which refers to the average rating of the user. *n* is the number of the common items between users *i* and *f*. In this method, the similarity value between *i* and *f* is ranging from [–1,1], the higher value means that the two users are more similar. The similarity method is applied two times, first for the explicit friends and second for implicit friends.

**Fig 2:**
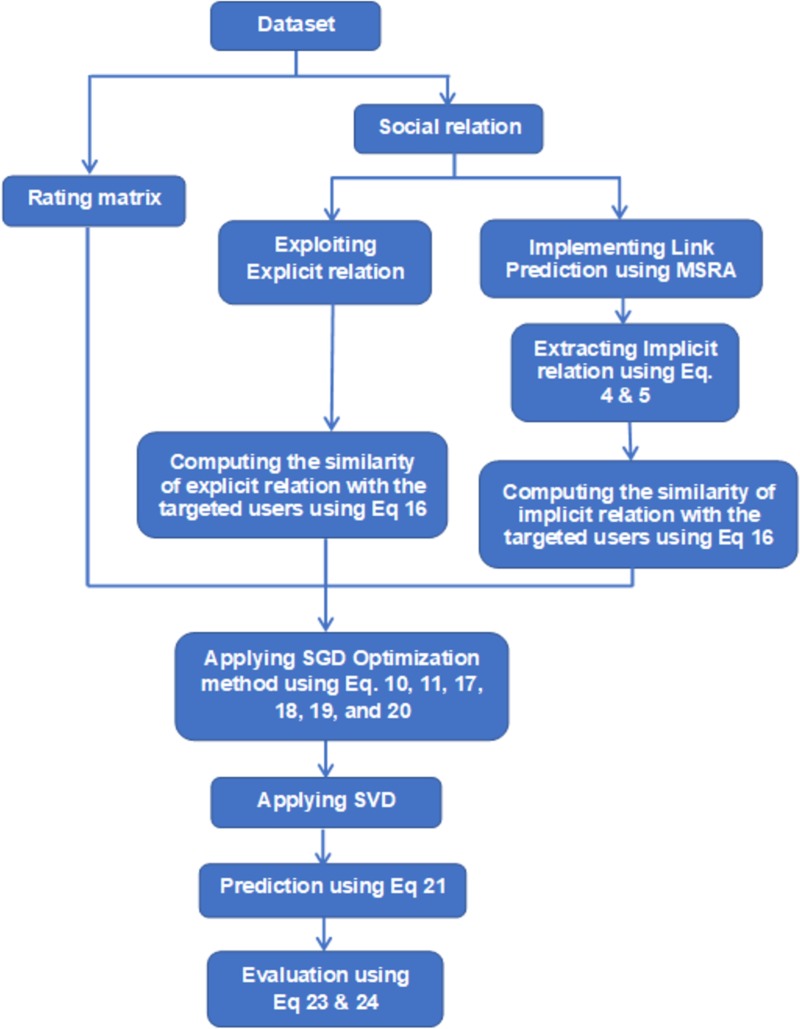
Overview of the proposed model.

After obtaining the similarity values, the user-item matrix with these values are ready to be used; now, all these resources are employed in SVD to produce the new model. According to Eqs (7) and (8) of the previous section, items are predicted via the SVD method without any external information such as social data. The prediction is also optimized according to Eq (15). In this model, this equation is modified to incorporate social information for optimizing the prediction to the following:

$$\frac{\partial E}{\partial U_{u}}=\sum_{(u,i)\in m}{\left(r_{ui}-\mu -b_{u}-b_{i}-V_{i}^{T}U_{u}\right)}^{2}+\lambda ({b_{i}}^{2}+{b_{u}}^{2}+{\left\|V\right\|}^{2}+{\left\|U\right\|}^{2})+\beta_{e}\sum_{i=1}^{u}\sum_{f\epsilon F(i)}sim(i,f){\left\|U_{i}-U_{f}\right\|}_{fro}^{2}+\beta_{i}\sum_{i=1}^{u}\sum_{f^{*}\epsilon F^{*}(i)}sim(i,f^{*}){\left\|U_{i}-U_{f^{*}}\right\|}_{fro}^{2}$$ (17)

$$\frac{\partial E}{\partial V_{i}}=\sum_{(u,i)\in m}{\left(r_{ui}-\mu -b_{u}-b_{i}-V_{i}^{T}U_{u}\right)}^{2}+\lambda ({b_{i}}^{2}+{b_{u}}^{2}+{\left\|V\right\|}^{2}+{\left\|U\right\|}^{2})$$ (18)

where *β*_*e*_ and *β*_*i*_ are constant values that are used to control the impact degrees of the explicit and implicit relationships, respectively; *F(i)* and F*(i) denote the numbers of explicit friends and implicit friends, respectively, of user *i*; and *sim(a*,*b)* refers to the similarity value between users a and b. Via the Stochastic Gradient Descent (SGD) optimization technique, regularization square error is reduced. The following Eqs 19 and 20 are used to update the factors U and V, Eq 21 is utilized to compute the prediction. (Fig 2), presents an overview of the proposed model.

$$U_{u}=U_{u}+\alpha \;(\frac{\partial U}{\partial E}V_{i}-\lambda U_{u})$$ (19)

$$V_{i}=V_{i}+\alpha \;(\frac{\partial V}{\partial E}U_{u}-\lambda V_{i})$$ (20)

$$r_{u,i}=\mu +b_{u}+b_{i}+V_{i}^{T}U_{u}+\lambda ({b_{i}}^{2}+{b_{u}}^{2}+{\left\|V\right\|}^{2}+{\left\|U\right\|}^{2})$$ (21)

## Experimental preparations

In this section, the datasets that are used in this study are described and the evaluation metrics are presented.

### Datasets description

Various datasets are utilized in RSs. In this study, two types of social network datasets are used: well-known datasets Last.Fm and Ciao. Last.fm is the United Kingdom online music system. Users can listen to several types of music, and it enables users to create friend relationships. Last.Fm was released in the HetRec workshop (Cantador et al. (2011) [41]) and includes 2100 users and 18745 items. The dataset contains the number of listens for each artist. Thus, the rating value is extracted via the same approach as was proposed by Juntao et al. [5], in which the items’ ratings (1–5) are computed by mapping the listening counts to integer values by applying as follows.

$$r=\left\{\begin{array}{c} \lfloor {log}_{10}l\rfloor +1,\;if\lfloor {log}_{10}l\rfloor +1\leq 5\\ 5,otherwise \end{array}\right\}$$ (22)

where *r* is the rating integer value, *l* denotes the listening count, and |*x*| denotes the operation of rounding towards zero.

In this paper, the number of users depends on the number of listening repetitions and all users have listened and rated 50 items. To compare our work with the EISR model, all users that have less than five relationships are removed. Thus, the final numbers of users and items are 1123 and 18745, respectively. The number of ratings in this sample is 55,140.

The second dataset that is used in this study is Ciao, which was gathered by Tang et al. (2012) [42] and contains 7375 users and 99746 items. Ciao is a product review website on which products can be rated and reviewed by users. Additionally, it enables users to establish social relations. In this study, all users with more than one item rating and at least one relation are chosen and the items with one or zero ratings are removed. After applying this condition, 6767 users with 22229 items remain. Table 1 presents the statistics of the two datasets.

**Table 1:** Statistics of the Last.Fm and Ciao datasets.

Dataset	Last FM	Ciao
**# users**	1123	6767
**# items**	18745	22229
**# rating**	55,140	185759
**Density**	0.0026	0.0012
**# explicit relation**	11064	111780
**# implicit relation using RAI**	166568	549490
**# implicit relation using MSRA**	177987	610710

### Evaluation metrics

Many matrices are used to evaluate the recommendation performance. Some of these matrices are used to compute top-N recommendations and others are used to evaluate the prediction accuracy. In this study, two accuracy functions are used: mean absolute error (MAE) and root mean square error (RMSE). These functions are well-known measures in the literature, according to Chen et al. [43]. MAE and RMSE are defined in the following formulas, respectively.

$$MAE=\frac{1}{N}\sum_{i=1}^{N}\left|r_{u,i}-{\bar{r}}_{u,i}\right|$$ (23)

$$RMSE=\sqrt{\frac{1}{N}\sum_{i=1}^{N}{\left(r_{u,i}-{\bar{r}}_{u,i}\right)}^{2}}$$ (24)

where *N* is the total number of predictions, *r*_*u*,*i*_ is the actual value in the dataset for item *i* that is specified by user *u*, and ${\bar{r}}_{u,i}$ is the result of the production system. If the values of MAE and RMSE are close to zero, the results are highly accurate, whereas if the values are large, the results are of low accuracy.

### Experimental setup

This implementation of this study depends on parameters for realizing the optimal results. These parameters are listed in Table 2.

**Table 2:** Parameter setup.

Dataset	Last FM	Ciao
β_e_ and β_i_	0.0002	0.001
**α**	0.09	0.01
λ_e_ and λ_i_	0.0001	0.0001
**K**	50	50
**Iterations**	5	5

The proposed model is benchmarked against state-of-the-art approaches; hence, we can determine whether our proposed method enhances the prediction performance. The following studies are utilized as benchmarks against our model:

- SVD [44] is used for comparison with the proposed method. Baseline SVD is utilized, which depends on the user-rating matrix without any extra information. It’s used for both datasets;
- PMF [45] is one of the most prevalent methods for dimension reduction. This method also uses only rating information and is used for Last.Fm only;
- Heats [46] is a heat-spreading algorithm that is used to enhance the prediction performance and is used for Last.Fm only;
- SR [31] stands for social recommendation. This method uses explicit social information along with the user-item matrix and is used as a baseline with Ciao and with the same result that is presented in [32];
- EISR-JC, which was proposed by Reafee et al. [32], stands for the explicit implicit social recommendation-Jaccard coefficient. It uses the implicit relations as supporting information to reduce the sparsity;
- EISR-CN, which was proposed by Reafee et al. [32], is utilized, in which CN abbreviates common neighbour. Implicit relations are also used;
- EISR-PA, which was also proposed by Reafee et al. [32], uses preferential attachment to exploit the implicit relations by applying similarity methods to predict a new relation;
- EISR-RAI, which was also proposed by Reafee et al. [32], uses the resource allocation index method to predict hidden relations in social information and can discover many relations. EISR-RAI is used for both datasets.

In the literature, the most similar model to that in our study is the EISR Reafee et al. [32] model, which has many versions. Thus, for fair comparison, the same conditions, such as the number of users, the explicit relationships for all users, the dimension of latent space, and the number of iterations, are used, as listed in Table 2. Incomplete cold start (ICS) users are considered in this study. In the Last.Fm dataset, 20% of all users are randomly selected. Each user in Last.Fm has 50 ratings; thus, the ratings of selected users are removed and only three ratings are retained for each ICS user. For the Ciao dataset, all users who have at most four ratings with at least one relationship are considered ICS users.

## Results and discussion

A 5-fold cross-validation technique is applied in this study. The datasets are divided randomly into five parts: four parts for training (80%) and one part for testing (20%). The cross-validation is computed five times and the average of the outcomes is regarded as a single valuation. Each scenario is implemented five times and the mean is computed to produce the final results. Tables 3 and 4 for all users and Tables 5 and 6 for cold-start users present the results in terms of MAE and RMSE. Each table consists of two functions for measuring the accuracy, namely, MAE and RMSE; the higher their values, the lower the accuracy. In addition, the proposed method is compared with eight approaches: the first three approaches utilize only the user-item matrix to predict missing ratings. By contrast, social recommendation (RS) uses two information sources: the user-item matrix and explicit social relationships. Moreover, EISR, which involves JC, CN, PA, and RAI, fuses the user-item matrix with explicit and implicit relationships.

**Table 3:** Evaluation performance on the Last.fm dataset for all users.

Metrics	Heats	PMF	SVD	SR	EISR-JC	EISR-CN	EISR-PA	EISR-RAI	Proposed method
**MAE**	0.4310	0.4253	0.4234	0.4214	0.4108	0.4068	0.4065	0.4048	**0.4016**
**RMSE**	0.5491	0.5339	0.5325	0.5301	0.5221	0.5210	0.5209	0.5201	**0.5174**

**Table 4:** Evaluation performance on the Ciao dataset for all users.

Metrics	SVD	SR (Baseline)	EISR-JC (Baseline)	EISR-CN (Baseline)	EISR-PA (Baseline)	EISR-RAI (Baseline)	Proposed method
MAE	0.7290	0.7280	0.7278	0.7275	0.7273	0.7271	**0.7266**
RMSE	0.9621	0.9608	0.9601	0.9598	0.9595	0.9591	**0.9576**

**Table 5:** Evaluation performance on Last.fm for cold-start users.

Metrics	SVD (Baseline)	SR (Baseline)	EISR-JC (Baseline)	EISR-CN (Baseline)	EISR-PA (Baseline)	EISR-RAI (Baseline)	Proposed method
MAE	0.4433	0.4317	0.4270	0.4217	0.4215	0.4210	**0.4194**
RMSE	0.5590	0.5493	0.5473	0.5464	0.5462	0.5457	**0.5438**

**Table 6:** Evaluation performance on Ciao for cold-start users.

Metrics	SVD (Baseline)	SR (Baseline)	EISR-JC (Baseline)	EISR-CN (Baseline)	EISR-PA (Baseline)	EISR-RAI (Baseline)	Proposed method
MAE	0.7748	0.7729	0.7680	0.7677	0.7665	0.7663	**0.7651**
RMSE	1.0241	1.0171	0.9922	0.9904	0.9897	0.9889	**0.9859**

According to Tables 3 and 4, the proposed method outperformed the previous approaches and the closest result to that of our method is that of EISR-RAI, with RMSE and MAE values of up to 0.0027 and 0.0032, respectively, for Last.fm and 0.0015 and 0.0005 for Ciao. Moreover, both SVD and PMF are dimension reduction techniques and both use the user-item matrix as the sole data source. According to Table 3, SVD outperforms the other approaches on PMF; this also demonstrated by [47].

Tables 5 and 6 present the results for cold-start users. The cold-start users are users with at most four ratings for each user and at least one relationship (since the core factor in the proposed method is social relationships). According to Tables 5 and 6, the proposed method outperforms the other methods in terms of MAE and RMSE, where the difference between the proposed method and closest study is 0.0019 in RMSE and 0.0016 in MAE for Last.FM and 0.0030 in RMSE and 0.0012 in MAE for the Ciao dataset. Hence, the proposed method predicts items with acceptable accuracy.

**Fig 3:**
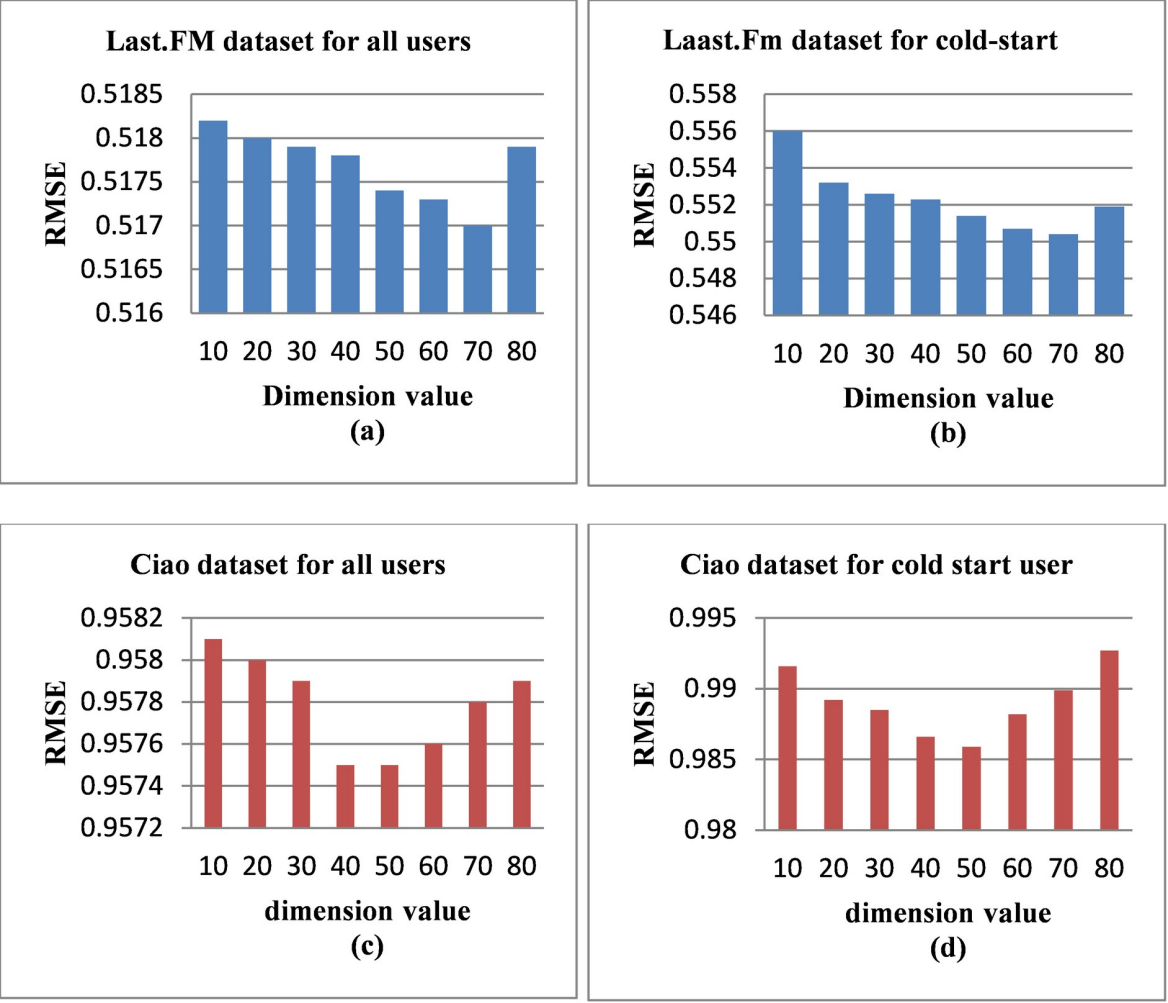
RMSE results for various numbers of dimensions.

According to the results of Tables 3 and 4 for all users and Tables 5 and 6 for cold-start users, the social relationships have a substantial impact on the results; for example, in Table 4, the difference between applying pure SVD and the best result in this paper (that of the proposed method) is 0.0045 in terms of RMSE. By contrast, in Table 6 (for the cold-start users), the difference between the same studies is 0.0382. The same is observed between Tables 3 and 5. Thus, using social information to compute the similarities of users to alleviate the incomplete cold-start user problem is more effective than using this information with users who have sufficient histories. Subsequently, the social information can support substantially the incomplete cold-start users by identifying the items that are rated by the friends of a targeted user and recommending these items to the user. By contrast, users who have sufficient histories the system can utilize their histories to find items that are similar to their tastes. Hence, the social information facilitates the prediction process for incomplete cold-start users and, to a lesser degree, for other users.

(Fig 3) presents the impact of changing the number of dimensions (*k*) on the results, the value of (*k*) shows the latent features dimension of the user-item matrix. In addition, when the value of (*k*) increases, more latent features will be included in the dimension reduction methods, subsequently the accuracy is improved. However, when the value of (*k*) increasingly continues, the accuracy will be affected negatively. As shown in Fig 3A and 3B plot the RMSE values for the Last.FM dataset; the RMSE values fluctuate as *(k*) is varied. When the dimension value (*k*) is 10, the RMSE is too high (low accuracy) comparing with other values. Moreover, the accuracy is increased as the dimension value increases and it attains its best result when the number of dimensions is 70 in both Fig 3A and 3B. However, in the same figures, the accuracy declines after 70 to approximately 0.5179 and to 0.5437 when the dimension value equals 80. Additionally Fig 3C and 3D plot the RMSE values for the Ciao dataset. The RMSE is improved as the number of dimensions is increased. The best result is attained when the number of dimensions equals 50, where the RMSE values are approximately 0.9575 and 0.9860 for Fig 3C and 3D, respectively. By contrast, when the number of dimensions is increased to more than 50, the accuracy decreases to 0.9580 and 0.9927 in Fig 3C and 3D, respectively. To sum up, when (*k*) raises, more latent features are added thus the accuracy is improved. Nonetheless, if (*k*) value passes the threshold, the accuracy decreases because extra features mean noise data.

## Conclusions

Cold-start and sparsity are widespread problems in RSs. In this study, a new model was proposed that exploits social information in the form of explicit and implicit relationships in addition to the user-item matrix. Regarding implicit relationships, MSRA was applied to predict hidden relations between users, which yielded meaningful information and facilitated the prediction process. Moreover, Pearson Correlation Coefficient algorithm was utilized to compute the similarity between explicit friends and implicit friends. Furthermore, user-item matrix and similarity values were incorporated in SVD method, which finds the best low-rank linear representation of the user-item matrix. Eventually, SGD algorithm was utilized to optimize the prediction. The proposed method was applied to two real datasets: Last.Fm and Ciao which applied for normal users and incomplete cold-start users. The results demonstrated that the proposed method outperforms eight state-of-the-art methods, namely, Heats, SVD, PMF, SR, EISR-JC, EISR-CN, EISR-CN, and EISR-RAI, in terms of accuracy. In addition, the results demonstrated that computing the similarity of social information with incomplete cold-start users has a stronger impact than using it with users that have reasonable ratings. Additionally, the proposed method displayed the impact of changing *k* value. When the value inflates, the accuracy is boosted, until reaching the threshold, afterwards the accuracy declines. Many issues remain to be further investigated future studies to enhance RS performance, such as finding a new method for computing the similarity between users. The implicit relationships can be exploited in several ways such as comments, opinion, tags. Moreover, another dimension reduction method can be applied to enhance the results. Finally, to develop this model, we plan to construct a new model that can handle the complete cold-start problem.

## Supporting information

S1 Data Last.FM dataset. (RAR)

S2 Data Ciao dataset. (RAR)
